# SCD2-mediated cooperative activation of IRF3-IRF9 regulatory circuit controls type I interferon transcriptome in CD4^+^ T cells

**DOI:** 10.3389/fimmu.2022.904875

**Published:** 2022-08-18

**Authors:** Toshio Kanno, Keisuke Miyako, Takahiro Nakajima, Satoru Yokoyama, Shigemi Sasamoto, Hikari K. Asou, Osamu Ohara, Toshinori Nakayama, Yusuke Endo

**Affiliations:** ^1^ Department of Frontier Research and Development, Laboratory of Medical Omics Research, Kazusa DNA Research Institute, Chiba, Japan; ^2^ Department of Applied Genomics, Kazusa DNA Research Institute, Chiba, Japan; ^3^ Department of Immunology, Graduate School of Medicine, Chiba University, Chiba, Japan; ^4^ Japan Agency for Medical Research and Development (AMED) - Core Research for Evolutional Science and Technology (CREST), AMED, Chiba, Japan; ^5^ Department of Omics Medicine, Graduate School of Medicine, Chiba University, Chiba, Japan

**Keywords:** CD4^+^ T cells, SCD2, fatty acid metabolism, IRF3, IFR9, RNA-seq, ChIP-seq

## Abstract

Type I interferons (type I-IFN) are critical for the host defense to viral infection, and at the same time, the dysregulation of type I-IFN responses leads to autoinflammation or autoimmunity. Recently, we reported that the decrease in monounsaturated fatty acid caused by the genetic deletion of *Scd2* is essential for the activation of type I-IFN signaling in CD4^+^ Th1 cells. Although interferon regulatory factor (IRF) is a family of homologous proteins that control the transcription of type I-IFN and interferon stimulated genes (ISGs), the member of the IRF family that is responsible for the type I-IFN responses induced by targeting of SCD2 remains unclear. Here, we report that the deletion of *Scd2* triggered IRF3 activation for type I-IFN production, resulting in the nuclear translocation of IRF9 to induce ISG transcriptome in Th1 cells. These data led us to hypothesize that IRF9 plays an essential role in the transcriptional regulation of ISGs in *Scd2*-deleted (sg*Scd2*) Th1 cells. By employing ChIP-seq analyses, we found a substantial percentage of the IRF9 target genes were shared by sg*Scd2* and IFNβ-treated Th1 cells. Importantly, our detailed analyses identify a unique feature of IRF9 binding in sg*Scd2* Th1 cells that were not observed in IFNβ-treated Th1 cells. In addition, our combined analyses of transcriptome and IRF9 ChIP-seq revealed that the autoimmunity related genes, which increase in patient with SLE, were selectively increased in sg*Scd2* Th1 cells. Thus, our findings provide novel mechanistic insights into the process of fatty acid metabolism that is essential for the type I-IFN response and the activation of the IRF family in CD4^+^ T cells.

## Introduction

While type I Interferon (type I-IFN) was initially discovered as a humoral factor with an important role in antiviral responses, dysregulated type I-IFN signaling has also been implicated in a number of inflammatory diseases including the regulation of progression in autoimmune diseases (1, 2). Interferon regulatory factors (IRF), which consist of nine members in mammals, are well-known transcription factors that regulate type I-IFN production and signal transduction (3, 4). IRFs play pivotal roles in the regulation of both innate and adaptive immune responses (3, 4). In particular, IRFs have been shown to be involved in the activation and differentiation of distinct immune cell populations (3, 4). Depending on which types of nucleic acid sensing pathway are triggered, different types of IRF family members are activated to control type I-IFN signaling (3, 4). STING and MAVS, which recognize intracellular DNA or RNA, respectively, activate IRF3 and IRF7 to induce the production of type I-IFN (2, 5). Also, a Toll like receptor-dependent activation of MyD88 induces the production of type I-IFN *via* IRF5 and IRF7 (6). In the context of signal transduction, IRF9 forms a complex called interferon stimulated gene factor 3 (ISGF3) with STAT1 and STAT2 to regulate the expression of interferon stimulated genes (ISGs) (3, 4, 7). The overproduction of type I-IFN and aberrant induction of ISGs are considered to be a major contributor to the pathology of autoimmune disease.

Although type I-IFN is mainly produced by innate immune cells, including plasmacytoid dendritic cells (pDCs) and macrophages, CD4^+^ T cells can also produce type I-IFN by recognizing cytosolic nucleic acids of foreign and autologous origin (8, 9). It has been reported that cGAMP, a potent STING ligand, activates IRF3 and IRF7 in CD4^+^ T cells, resulting in the type I-IFN mediated inhibition of cGAMP- induced growth inhibition (8). CD4^+^ T cells infected with HIV or HSV also produce type I-IFN to induce anti-viral responses (9). Due to the ubiquitous expression of the type I-IFN receptor (IFNAR), most cells can receive type I-IFN signaling and increase the expression levels of ISGs. In addition to the upregulation of ISGs, type I-IFN has been shown to influence the ability of CD4^+^ T cells to construct follicular helper T cells, which help B cells produce antibodies upon pathogen encounters. It has also been reported that type I-IFN regulates the cytokine production of CD4^+^ T cells in experimental autoimmune uveitis, a pathological model of autoimmune disease, and contributes to the regulation of the disease (10).

Intracellular metabolism has been reported to contribute to the regulation of T cell proliferation, activation, and memory generation. In naïve CD8^+^ T cells, a metabolic switch from oxidative phosphorylation (OXPHOS) to glycolysis is required for effector differentiation upon antigen entry (11). Especially, cell intrinsic lipolysis is essential for the generation and maintenance of memory CD8^+^ T cells (12). CD4^+^ T cells also require lipid metabolism for their proliferation, activation, and memory generation (13, 14). The homeostasis of regulatory T cells in lung tissue relies on the metabolism of Acsbg1-regulated acyl-coenzyme A (CoA) (15). Our recent study also indicates that ACC1, a rate limiting enzyme of fatty acid biosynthesis, is an essential metabolic regulator of CD4^+^ T cell-mediated allergic inflammation in the lung and skin (16).

It has been suggested that the lipid metabolism and type I-IFN responses are mutually connected. Recent studies have reported that cellular lipid metabolism regulates type I-IFN responses (17). Bensinger et al. showed that altering the balance between cholesterol biosynthesis and scavenging, rather than reducing the endogenous lipid pool size, is essential for achieving type I-IFN responses in macrophage (17). We also previously found that genetic deletion or pharmacological inhibition of acetyl-CoA carboxylase (ACC1), which is rate limiting enzyme of fatty acid biosynthesis, substantially increases the basal expression of ISGs *via* the spontaneous production of type I-IFN (18). Our data further indicated that selective decrease in monounsaturated fatty acid (MUFA) biosynthesis is crucial for the induction of type I-IFN response in CD4^+^ T cells (18). Indeed, CRISPR/Cas9-mediated genome editing of the stearyl-CoA desaturase 2 (Scd2), which is responsible for MUFA biosynthesis, dramatically increased the expression of ISGs in CD4^+^ T cells (18). On the other hand, type I-IFN signaling changes the fatty acid oxidization, OXPHOS and expression levels of genes related to lipid metabolism in pDCs and macrophages (19). Although the relationships between lipid metabolism and the type I-IFN response have been investigated, the types of IRF family that are important for the lipid metabolism-mediated type I-IFN response remain unclear.

In the present study, using a combination of global RNA-sequencing and ChIP-sequencing technologies, we investigated the critical IRF family members for the type I-IFN response induced by the suppression of the MUFA biosynthesis pathway. Enhanced type I-IFN signaling in *Scd2* KO Th1 cells (hereafter referred to as sg*Scd2* Th1 cells) highly depend on IRF3 and IRF9 activation, but not IRF7. The genetic deletion of *Scd2* caused nuclear translocation of IRF3 and IRF9 to induce type I-IFN production and ISG transcription, respectively. Using a ChIP-seq analysis, we also revealed genome wide binding profiles of IRF9 in sg*Scd2* Th1 cells and found that IRF9 bound to the regulatory region of ISGs, the expression levels of which are characteristically elevated in patients with autoimmune diseases. Our results clarified the mechanistic insight into the regulatory relationship between lipid metabolism and the IRF family in the type I IFN response.

## Material and methods

### Mice

C57BL/6 mice were purchased from CLEA Japan. All mice were used at 6–8 weeks old and were maintained under specific-pathogen-free conditions. Almost equal number of male and female animal was used for this study. Animal care was conducted in accordance with the guidelines of KAZUSA DNA research institute, Japan.

### Cell preparation

Splenic naïve CD4^+^ T cells were obtained by the negative selection using the Mojo Sort Mouse CD4 T Cell Isolation Kit (Biolegend #480006) and positive selection using CD62L MicroBeads, mouse (Miltenyi Biotec #130-049-701). Naïve CD4^+^ T cells were plated onto 24-well tissue culture plates (Costar #3526) pre-coated with 1μg/ml Anti-mouse TCRβ (clone H57-597, Biolegend #109255, Lot#B312356) with 1μg/ml anti-CD28 antibody (clone 37.51, BioLegend #102116, Lot#B346685). Th1 cell cultures contained 15 ng/ml IL-2 (Peprotech #212-12, Lot#067108), 10 ng/ml recombinant mouse IL-12 (WAKO #095-05331, Lot#SAQ1865) and 1μg/ml anti-IL-4 antibody (Biolegend #504122, Lot#B336348). Recombinant mouse IFNβ (Biolegend #581302, Lot#B229335) were dissolved in PBS supplemented with 3% BSA and treated with 100U/ml. IFNAR neutralizing antibody (clone MAR1-5A3, Biolegend #127322, Lot#B163670) was treated with 10μg/ml.

### Cas9 mediated-genome-editing

The short guide RNA (sgRNA) was designed using the online tool provided by CHOPCHOP 3.0.0 (http://chopchop.cbu.uib.no) (20). sgRNA were purchased from FASMAC. Freshly isolated naïve CD4^+^ T cells were activated with plate bound anti-CD3 and CD28 antibodies for 24 hours as described in section of Cell Preparation. 24 h after T cell activation, these cells were electroporated with a Neon transfection kit and device (Thermo Fisher Scientific #MPK1025). Briefly, Cas9 proteins (Takara #632641) were prepared immediately before experiments by incubating 1μg Cas9 with 0.3μg sgRNA (FASMAC #GE-001) in transfection buffer at room temperature for 10 min. Then, mixture of Cas9 and sgRNA was added with 1 μl of Cas9 electroporation enhancer (Integrated DNA Technologies #1075916). The electroporation was carried out following parameter (1800 Volts of pulse voltage, 10 ms of pulse width, and 3 pulses). 24 hours after the electroporation, cells were cultured for 3 days without TCR-stimulation and used as experimental samples.

### Primer sequences for Cas9 mediated-genome-editing

Control-targeting: 5’-CGTATTACTGATATTGGTGGG-3’sg*Scd2*: 5’-AACCAGTGTGATCCCGTACAAGG-3’ sg*Irf3*: 5’-GAAGGGCCTGAGGTCGAACACGG-3’ sg*Irf7*: 5’-TCAGCAGCGGCCAGTACGAGGGG-3’ sg*Irf9*: 5’-TACGCTGCACCCGAAAGCTGCGG-3’

### Quantitative real-time PCR

Total RNA was isolated with the TRIzol reagent (Invitrogen #15596-018). cDNA was synthesized with an oligo (dT) primer and Superscript II RT (Invitrogen #18064-014). Quantitative RT-PCR was performed using TB Green Real Time PCR kit (Takara #RR820A). Primers were purchased from Thermo Fisher Scientific. Gene expression was normalized with the Hprt mRNA signal or the 18S ribosomal RNA signal.

### Primer sequences for real-time PCR

18S_FW: 5’-AAATCAGTTATGGTTCCTTTGGTC-3’ 18S_RV: 5’-GCTCTAGAATTACCACAGTTATCCAA-3’ *Hprt*_FW: 5’-TCCTCCTCAGACCGCTTTT-3’ *Hprt*_RV: 5’-CCTGGTTCATCATCGCTAATC-3’ *Atp8b4*_FW: 5’-GAAGGAGGGAGAAACCAGGC-3’ *Atp8b4*_RV: 5’-TGGTGGTGAACCATGTCAGG-3’ *Cd47*_FW: 5’-GCTTCTGGACTTGGCCTCAT-3’ *Cd47*_RV: 5’-CCTCTGGTTGGAAGCGACAA-3’ *F830016B08Rik*_FW: 5’-AGCCTGGAGCACTGTAAAGG-3’ *F830016B08Rik*_RV: 5’-AGGAGCTGACCCATGTTGATG-3’ *Gbp7*_FW: 5’-AATCCGGTGCAGGCTGGTTA-3’ *Gbp7*_RV: 5’-ACTGTGGTGCCCAGATTGAA-3’ *Gbp9*_FW: 5’-ACTTGGACCTGTGCTGTGG-3’ *Gbp9*_RV: 5’-CACATCCAGATGCCCTTGGT-3’ *Herc6*_FW: 5’-TCTGGCATCTTTAACTTTGATGC-3’ *Herc6*_RV: 5’-TGAAAACAACCATATCTGAGGATTC-3’ *Ifi203*_FW: 5’-AGTCTCCCCAGGAAGACAGC-3’ *Ifi203*_RV: 5’-TTGTCCTCAATCCAGTCCGC-3’ *Ifit3b*_FW: 5’-TTCCCAGCAGCACAGAAACA-3’ *Ifit3b*_RV: 5’-TCAGCTTGCCCTAAGCACTC-3’ *Irgm2*_FW: 5’-GAGCAGGGTCTGAGAGGAAAC-3’ *Irgm2*_RV: 5’-TTGTCGAGCAACGGGGCAA-3’ *Irf7*_FW: 5’-CTTCAGCACTTTCTTCCGAGA-3’ *Irf7*_RV: 5’-TGTAGTGTGGTGACCCTTGC-3’ *Oas2*_FW: 5’-AGTGACATGGTGGGAGTGTTC-3’ *Oas2*_RV: 5’-CTTCCGGGGGTCTGCATTAC-3’ *Oasl1*&FW: 5’-GGCCAACCAGTGTCTGAAA-3’ *Oasl1*&RV: 5’-TGGATATCGGGTGCTCTCTT-3’ *Samhd1*_FW: 5’-CAAGCGGTCAGGATCAATAAA-3’ *Samhd1*_RV: 5’-TGAGCTGCTCTGCAAATTTCT-3’ *Tlr7*_FW: 5’-TGGCTCCCTTCTCAGGATGA-3’ *Tlr7*_RV: 5’-ATGTCTCTTGCTGCCCCAAA-3’ *Tmem184b*_FW: 5’-CCTCAGTGCAGTGGCTTTGA-3’ *Tmem184b*_RV: 5’-CCCTCACTGTCATGGTTCCC-3’ *Trim21*_FW: 5’-GGGAAAGAGTTGGCCGAGAA-3’ *Trim21*_RV: 5’-ACCACGAATCCTCCTCTCCA-3’ *Trim26*_FW: 5’-CTGCACTACACAGGACACCA-3’ *Trim26*_RV: 5’-TGTAGGTATCCACTGGCCGA-3’

### Primer sequences for ChIP qRT-PCR

*Ifit1* (Promoter)_FW: 5’-TGGCAGGGATGTCTCACTCT-3’ *Ifit1* (Promoter)_RV: 5’-GAAGGCTCTGAAACGGATACA-3’ *Irf7* (Promoter)_FW: 5’-TGAGGTTTGAGAACTTGTGGTC-3’ *Irf7* (Promoter)_RV: 5’-TCCCGCTACATCTGTAGTCACA-3 *Il5* (Promoter)_FW: 5’-AAGTCTAGCTACCGCCAATA-3’ *Il5* (Promoter)_RV: 5’-AGCAAAGGTGAGTTCAATCT-3 *Va* (Promoter)_FW: 5’-TCCATACAGTTCCTGCAGTAGCTG-3’ *Va* (Promoter)_RV: 5’-CATCTCCCCAACCCCAAGATATA-3

### FACS analysis

Dead cells were first stained with Fixable Viability Dye eFluor 780 (1:1000, eBioscience #65-0865-14) for 10 min. For pSTAT1, pIRF7 and IRF7 staining, sample preparation was conducted with Lyse/Fix buffer for 10 min at 37°C (BD Biosciences #558049) and Perm buffer III (BD Biosciences #558050) for 30 min on ice according to the manufacture’s protocol. Cells were stained with pSTAT1-PE (1:50, Clone 4a, BD Biosciences #562069), anti-pIRF7 Alexa647 (1:200, Clone K47-61, BD Biosciences #558630) and anti-IRF7 PE (1:200, Clone MNGPKL, eBioscience #12-5829-82) for 45 min in the dark. Flow cytometric data were analyzed after removal of dead cells and doublets cells with Flowjo software (version 10.4).

### Immunoblotting

Immunoblotting was performed as described previously (21). Briefly, cytoplasmic extracts and nuclear extracts were prepared using NE-PER Nuclear and Cytoplasmic Extraction Reagent (Thermo Fisher Scientific #78833) containing protease inhibitor cocktail (Thermo Fisher Scientific #87785) and phosphatase inhibitor cocktail (Thermo Fisher Scientific #78420). Protein concentrations were determined using a Bradford protein assay (BIORAD # 5000006JA) and equal amount of sample protein was separated by SDS-PAGE and then transferred onto a PVDF membrane (GE Healthcare #10600023). After blocking with protein-free blocking buffer (Thermo Fisher Scientific, #37584), the membranes were incubated with the appropriate antibody concentration. HRP-conjugated anti-rabbit IgG (GE Healthcare #NA934) and HRP-conjugated anti-mouse IgG (1:2000, GE Healthcare #NA931) were used as the secondary antibodies. The antibodies used for the immunoblot analysis were anti-SCD2 (1:1000, #sc-518034), anti-phosphoIRF3(Ser396) (1:2000, CST#4947), anti-IRF3 (1:2000, Biolegend#655701), anti-IRF9 (1:2000, CST#28845), anti-STAT1 (1:2000, CST#14995), anti–phosphoSTING (Ser365) (1:2000, CST#72971S), anti–STING (1:2000, CST#13647S), anti–phosphoTBK1 (S172) (1:2000, CST#5483S), anti–TBK1 (1:2000, CST#3504S) and anti-Tubulin (1:2000, Thermo Fisher Scientific#14-4502-82).

### 3′mRNA-seq library preparation

TRIzol reagent (Thermo Fisher Scientific #15596-018) was used for the extraction of total cellular RNA and Quantus Fluorometer (Promega #E6150) was used for determining of RNA concentrations. Total 500 ng of RNA was used for the 3′mRNA library preparation with QuantSeq 3′ mRNA-Seq Library Prep Kit FWD (LEXOGEN #015.384) according to the manufacture`s protocol. After the PCR step, size distribution and yield of the library was determined by the D1000 high sensitivity tape station (Agilent #5067-5582) or Agilent High Sensitivity DNA kit on the bioanalyzer (Agilent #5067-5583). The pooled libraries were loaded on the Illumina Nextseq500 platform and analyzed by 75bp single read.

### Analysis of RNA-seq data

Adaptor sequences were trimmed from the raw RNA-seq reads with fastp (v 0.23.1) (22). Trimmed reads of each sample were mapped to the reference mouse genome mm10 by using STAR (v 2.3.1) (23) and normalized to 1 million reads in the original library. Genes with an average of 10 or more reads in either group were subjected for further analysis. 1.5-fold changed genes was defined as differentially expressed genes. PCA analysis and heatmap were depicted with R software (https://cran.r-project.org/) (v 3.6.0).

### ChIP qRT-PCR

1 × 10^7^ cells were fixed in 1% formaldehyde at 25°C for 10 min, followed by the addition of 1.25 M glycine. Cells were sedimented, washed, and lysed with lysis buffer (50mM HEPES (pH7.9), 140mM NaCl, 1mM EDTA (pH8.0), 10% Glycerol, 0.5% NP-40, 0.25% TritonX-100, 1 mM PMSF, 1 mg/ml aprotinin, and 1 mg/ml leupeptin). The lysates were sonicated to reduce the DNA lengths to between 200 and 1,000 bp using Covaris (M&S Instruments Inc. #M220). The soluble fraction was diluted in ChIP dilution buffer and incubated with Ab conjugated with Dynabeads proteins A and G (VERITAS #DB10015) overnight at 4°C. The immune complexes were then captured using a magnet and washed with low-salt, high-salt, LiCl, and Tris-EDTA wash buffer. Enriched chromatin fragments were eluted with elution buffer (0.1 M NaHCO3 containing 1% SDS). The eluted material was incubated at 65°C for 6 h to reverse the formaldehyde cross-links and treated with RNase A (10 mg/ml) and proteinase K (40 mg/ml). DNA was extracted with a QIAquick PCR purification kit (Qiagen #28104). The total input DNA (cellular DNA without immunoprecipitation) was purified in parallel. The antibody used in the ChIP assay was as follows: anti-IRF9 (CST#28845). Quantitative RT-PCR was performed using TB Green Real Time PCR kit (Takara #RR820A). Enrichment was calculated with the following formula: specific antibody ChIP/input DNA. Primers were purchased from Thermo Fisher Scientific.

### ChIP-seq library preparation

ChIP was performed as described in “ChIP qRT-PCR&". Library preparation was conducted with NEBNext Ultra II DNA Library Prep Kit (New England Biolabs #E7645L) for Illumina according to manufactured protocol. After PCR step, size distribution and yield of the library was determined by Agilent High Sensitivity DNA kit on the bioanalyzer (Agilent #5067-5583). qRT-PCR was conducted for determining of library concentration using GenNext NGS Library Quantification Kit (TOYOBO #NLQ-101). The pooled libraries were loaded on the Illumina Nextseq500 platform and analyzed by 75bp single read.

### Analysis of ChIP-seq data

Adaptor sequences were trimmed from the raw ChIP-seq reads with fastp (v 0.23.1). Trimmed reads of each sample were mapped to the reference mouse genome mm10 by using Bowtie2 (v 0.12.8) (24) and peak call was used with MACS2 (v 2.1.2) (25). Generated file was used for depicting heatmap and PCA analysis with DiffBind (v 3.15) (26). BED files recording ChIP-seq signals were converted to BiGWig files using deeptools (v 3.5.0) (27) and normalized to reads per genome coverage. The integrative Genomic Viewers software program (v 2.4.1) (28) was used for visualization of BiGWig files. HOMER tag directories, which were created by the HOMER platform (v 4.1.0) (29) from the aligned Sequence Alignment/Map (SAM) formats using SAMtools (v 1.15.1) (30). When ChIP peaks were annotated to the promoter of the closest gene, HOMER annotatePeaks.pl (mm10 genome build) was used. The tag count density was normalized as tags per 10 million reads in the original library.

### Statistics and reproducibility

Data are expressed as mean ± SD. The data were analyzed with the Graphpad Prism software program (version 7). Differences were assessed using unpaired two-tailed student t tests or one-way anova followed by tukey’s multiple comparisons test. Differences with P values of <0.05 were considered to be significant. No data were excluded from the analysis of experiments. Mice were commercially sourced and randomized into experimental groups upon arrival, and all animals within a single experiment were processed at the same time. For RNA-sequencing analysis and ChIP-sequencing analysis, the investigator was blinded. Data display similar variance between groups and are normally distributed where parametric tests are used.

## Results

### Genetic deletion of SCD2 triggered cooperative activation of IRF3 and IFR9 to induce ISGs in T cells

**Figure 1:**
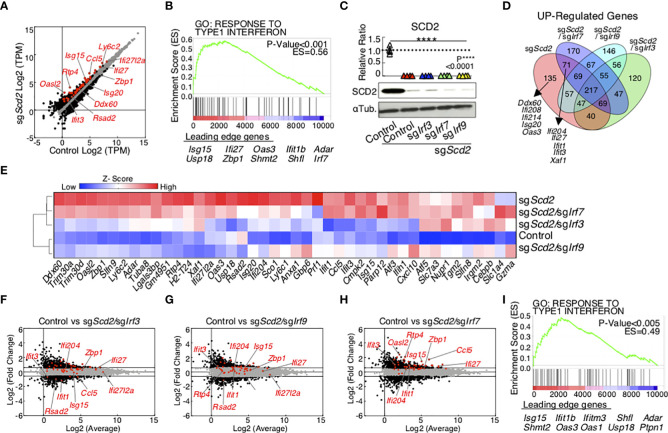
The transcriptome of ISGs in sgScd2 Th1 cell depends on IRF3 and IRF9, but not IRF7. **(A)** A scatter plot of gene expression by RNA-sequencing (n = 5 per sample) compares in control and sgScd2 Th1 cells. The genes with over 1.5-fold changes are marked with black dots, and ISGs are marked with red dots (Control, n = 5; sgScd2, n = 5 biologically independent sample). **(B)** GSEA reveals the upregulation of the ISGs in sgScd2 Th1 cells. Genes are ranked into an ordered list on the basis of fold change in control and sgScd2 Th1 cells. Genes below the picture indicate leading edge subset. (**C**) Western blot analysis of SCD2 from control, sgScd2, sgScd2/sgIrf3, sgScd2/sgIrf7 and sgScd2/sgIrf9 Th1 cells. The summary of relative intensity was shown. Band intensity was determined by image j. **(D)** The Venn diagram showed overlaps and differences between 1.5-fold increased genes in sgScd2, sgScd2/sgIrf3, sgScd2/sgIrf7 or sgScd2/sgIrf9 Th1 cells compared to control Th1 cells. **(E)** A heat map depicts the gene relevant to **(B)**. **(F–H)** A scatter plot of gene expression by RNA-sequencing (n = 5 per genotype) compares sgScd2/sgIrf3 **(F)**, sgScd2/sgIrf9 **(G)** or sgScd2/sgIrf7 **(H)** Th1 cells against control Th1 cells. **(I)** GSEA reveals the upregulation of the ISGs in sgScd2/sgIrf7 Th1 cells. Genes are ranked into an ordered list on the basis of fold change in control and sgScd2/sgIrf7 Th1 cells. Genes below the picture indicate leading edge subset. Three experiments were performed and showed similar results **(C)**. Data represent mean ± SD (one-way ANOVA test followed by Tukey’s post-hoc test for multiple comparisons, P****<0.0001).

Previously we reported that the suppression of monounsaturated fatty acid (MUFA) biosynthesis increased the expression levels of ISGs in CD4^+^ T cells (18). However, it is still unclear which types of IRF family are crucial for the MUFA-mediated control of ISGs in T cells. Among the IRF family members, IRF3, 7 and 9 have been well studied for the regulation of interferon production in innate immune cells; however, the role of these factors remains poorly understood in T cells (34). Therefore, we first performed CRISPR/Cas9-mediated genome editing of *Irf3*, *Irf7* or *Irf9* in sg*Scd2* T cells, and analyzed the global gene expression profiles of each group using RNA-sequence analyses. Consistent with our previous data, the genetic deletion of *Scd2* induced the expression of ISGs in Th1 cells (**Figure 1A**). A gene set enrichment analysis (GSEA) showed statistically significant enrichment of type I-IFN inducible genes in sg*Scd2* Th1 cells (**Figure 1B, C** and **Supplementary Figure 1A**). To evaluate the role of *Irf3*, *Irf7* or *Irf9* in the regulation of ISGs in sg*Scd2* Th1 cells, we next performed simultaneous knock out of *Scd2* and those of IRF genes (**Supplementary Figure 1B**). RNA-seq showed that the gene expression profile of sg*Scd2*, sg*Scd2*/sg*Irf3*, sg*Scd2*/sg*Irf7 or* sg*Scd2*/sg*Irf9* Th1 cells was largely altered in comparison to control Th1 cells (**Supplementary Figure 1B**). Among them, the double knockout of *Scd2* with *Irf7* very mildly affected the gene expression profiles and showed a relatively closer pattern to *Scd2* single-deficient Th1 cells. The Venn diagram revealed that overlapped genes between sg*Scd2* Th1 cells or sg*Scd2*/sg*Irf7* Th1 cells were involved in type I-IFN inducible genes which were found by GSEA (**Figure 1D**). A hierarchical clustering heatmap and a principal component analysis (PCA) revealed that the ISG expression profiles of sg*Scd2*/sg*Irf3* or sg*Scd2*/sg*Irf9* Th1 cells were very close to the phenotype of control Th1 cells (**Figure 1E** and **Supplementary Figure 1C**). We also found that sg*Scd2*/sg*Irf3* and sg*Scd2*/sg*Irf9* Th1 cells failed to increase the expression of ISGs (**Figures 1F, G**). On the other hand, sg*Scd2*/sg*Irf7* Th1 cells still possess the ability to induce the expression of ISGs (**Figure 1H**). Importantly, the GSEA also revealed the significantly increased transcription of genes related to type I-IFN inducible pathways in sg*Scd2*/sg*Irf7* Th1 cells (**Figure 1I**, sg*Scd2*/sg*Irf3*: p>0.o5, sg*Scd2*/sg*Irf9*: p>0.05). Consistent with the results of the GSEA, a gene ontology analysis also showed that the accumulation of ISGs is no longer observed in sg*Scd2*/*Irf3* or sg*Scd2*/*Irf9* Th1 cells (**Supplementary Figures 1D–G**). Taken together, these data indicate that IRF3 and IRF9, but not IRF7, are responsible for the induction of ISGs when the Scd2-MUFA metabolism is targeted in Th1 cells.

### Genetic deletion of *Scd2* induced nuclear translocation of IRF3 and IRF9 to control the l type I-IFN signaling pathway

**Figure 2:**
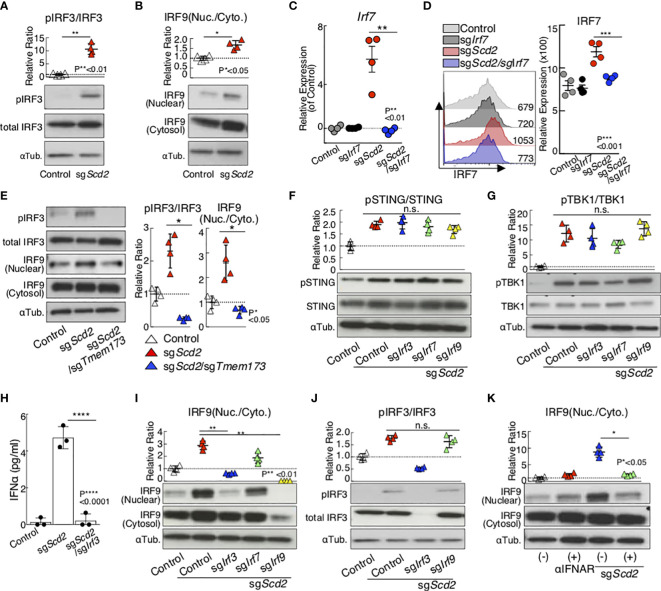
Gene deletion of SCD2 resulted in the translocation of IRF3 and IRF9 from cytosolic to nuclear. **(A, B)** Western blot analysis of phospho-IRF3 (pIRF3), total IRF3 **(A)** and IRF9 **(B)** from control and sgScd2 Th1 cells. **(C)** qRT-PCR analyses of the relative expression of Irf7 from control, sgIrf7, sgScd2 and sgScd2/sgIrf7 Th1 cells. Relative expression (normalized to Hprt) with SD is shown. **(D)** Intracellular staining and flow cytometry analyzing of IRF7 in control, sgIrf7, sgScd2 and sgScd2/sgIrf7 Th1 cells. Mean fluorescence intensity (MFI) of IRF7 are shown. Summary data of three independent experiments of IRF7 expression are shown here. Each dot represents one experiment. Data are means ± SD. (n = 3 per each group biologically independent sample). (E) Western blot analysis of pIRF3, total IRF3 and IRF9 from control, sgScd2 and sgScd2/sgTmem173 Th1 cells. (F, G) Western blot analysis of pSTING, total STING **(F)** and pTBK1, TBK1 **(G)** from control, sgScd2, sgScd2/sgIrf3, sgScd2/sgIrf7 and sgScd2/sgIrf9 Th1 cells. **(H)** The amount of IFNα in the cell supernatant was measured by ELISA. Data are means ± SD. **(I)** Western blot analysis of IRF9 from control, sgScd2 and sgScd2/sgTmem173 Th1 cells. **(J)** Western blot analysis of pIRF3 and total IRF3 from control, sgScd2, sgScd2/sgIrf3 and sgScd2/sgIrf9 Th1 cells. **(K)** Western blot analysis of IRF9 from sgScd2 Th1 cells treated with 10μg/ml IFNAR neutralizing antibody. Isotype antibody was used as control. Band intensity was determined by image j and summary of three independent experiments was shown. Three technical replicates were performed with quantitative RT-PCR and relative expression (normalized to Hprt) with SD is shown **(C)**. Three experiments were performed and showed similar results. Data represent mean ± SD (unpaired two-tailed student t tests or one-way ANOVA test followed by Tukey’s post-hoc test for multiple comparisons, P*<0.05, P**<0.01, P***<0.001, P****<0.0001). ns means “not significant”.

Next, we performed a molecular analysis to examine the functional requirements of IRF3 and IRF9 to control type I-IFN signaling in sg*Scd2* Th1 cells. The genetic deletion of *Scd2* induced phosphorylation of IRF3, which is a marker of activated IRF3 (**Figure 2A** and **Supplementary Figure 2A**). Furthermore, we also confirmed the moderate translocation of IRF9 into the nuclei in sg*Scd2* Th1 cells compared with phosphorylation of IRF3 (**Figure 2B** and **Supplementary Figure 2B**). Although the genetic deletion of *Scd2* increased the IRF7 mRNA and protein expression levels (**Figures 2C, D** and **Supplementary Figure 2C**), the level of IRF7 phosphorylation was unchanged between control and sg*Scd2* Th1 cells (**Supplementary Figure 2D**). Previously, we reported that the activation of the STING-TBK1 axis is needed to induce the expression of ISGs by the genetic deletion of *Scd2* in Th1 cells (18). Consistent with this observation, we found that the double knockout of *Scd2* and *Tmem173*, which encodes STING, failed to activate IRF3 and IRF9 (**Figure 2E** and **Supplementary Figures 2E**). Even when *Irf3* or *Irf9* were deleted in sg*Scd2* Th1 cells, higher levels of phospho-STING and phospho-TBK1 were still detected (**Figures 2F, G** and **Supplementary Figures 2F, G**). These data indicate that IRF3 and IRF9 can be activated in the downstream molecules of the STING pathway in sg*Scd2* Th1 cells. Furthermore, the spontaneous production of IFNα is heavily dependent on IRF3 in sg*Scd2* Th1 cells (**Figure 2H**). We also found that sg*Scd2*/sg*Irf3* Th1 cells failed to induce the nuclear translocation of IRF9 (**Figure 2I** and **Supplementary Figure 2H**). In contrast, the level of phospho-IRF3 was not affected in sg*Scd2*/sg*Irf9* Th1 cells (**Figure 2J** and **Supplementary Figure 2I**). Blockade of the type I-IFN receptor suppressed the nuclear translocation of IRF9, indicating that extrinsic type I-IFN was required to induce IRF9 to translocate into nuclear fraction (**Figure 2K** and **Supplementary Figure 2J**). Taken together, these data indicated that the deletion of *Scd2* triggered IRF3 activation for type I IFN production, resulting in the nuclear translocation of IRF9 to induce the ISG transcriptome.

### Genome-wide comparison of IRF9 occupancy between *Scd2*-deleted and IFNβ-treated Th1 cells

**Figure 3:**
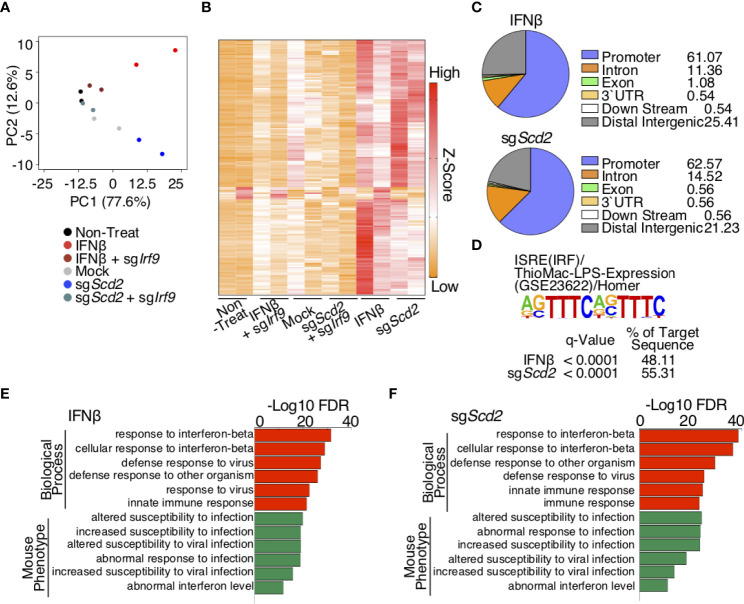
IRF9 ChIP-seq analysis revealed similar genome wide binding profile of IRF9 in sgScd2 Th1 cells IFNβ-treated Th1 cells. **(A)** Projections of PC1 and PC2 for normalized IRF9 ChIP-seq signal intensities of derived from non-treat, IFNβ, IFNβ-treated sgIrf9, mock, sgScd2 and sgScd2/sgIrf9 Th1 cells. Duplicates of each group are shown (ChIP-seq: PC1 77.6%, PC2 12.6%). **(B)** A heat map depicts the normalized IRF9 ChIP-seq signal intensities relevant to **(A)**. **(C)** Pie chart showed the distribution and genomic location of transcription factor IRF9, showing the percentage for each genomic location category in IFNβ-treated and sgScd2 Th1 cells. **(D)** Results of the known motif enrichment analysis de novo motif analysis from IFNβ-treated and sgScd2 Th1 cells. **(E, F)** GREAT analysis of IRF9 ChIP-seq peaks in IFNβ-treated **(E)** and sgScd2 **(F)** Th1 cells. The enriched terms for GO Biological Process and mouse phenotype are shown.

Our data indicated that the genetic deletion of *Scd2* enhanced the spontaneous production of type I-IFN, resulting in tonic type I-IFN stimulation and the nuclear translocation of IRF9. These data led us to hypothesize that IRF9 plays an essential role in the transcriptional regulation of ISGs in sg*Scd2* Th1 cells. To address this point, we assessed the genome-wide binding pattern of IRF9 using *Scd2*-deleted or type I-IFN stimulated Th1 cells by chromatin immunoprecipitation coupled with high-throughput DNA sequencing (ChIP-seq). To more accurately evaluate the IRF9 binding profiles, we prepared IRF9-deficient Th1 cells as a negative control. In order to identify target genes, we first conducted peak calls with MACS2 (25). A ChIP qRT-PCR analysis also confirmed that the binding of IRF9 to promoter regions of ISGs, including *Ifit1* or *Irf7*, was repeatedly detectable in type I-IFN treated or sg*Scd2* Th1 cells (**Supplementary Figures 3A, B**). Importantly, only lower signal intensity was observed in negative control regions, including *Il5* or BCR variable (*Vα*) regions (**Supplementary Figures 3C, D**). We identified 38, 141, 13 and 185 peaks for IRF9 in mock-control, *Scd2*-deleted, non-treated and IFNβ-treated Th1 cells respectively (**Supplementary Figures 3E, F**). To enumerate IRF9-bound genes, genes for which at least one significant peak (4-fold increase in signal intensity in comparison to input DNA) had been detected in the gene locus were selected (hereafter referred to as IRF9-bound genes). Each gene locus was defined as a region from 3 kb upstream of the transcriptional start site to -5 kb downstream of the transcriptional end site. We used a PCA and signal heat map to visualize the binding profiles of IRF9 in each cell population (**Figures 3A, B**). PCA revealed that a cluster of sg*Scd2* Th1 cells was located close to that of IFNβ-treated Th1 cells and was distinct from Mock and sg*Scd2*/sg*Irf9* Th1 cells (**Figure 3A**). Moreover, a heat map analysis showed a strong signal intensity in the groups of IFNβ-treated or sg*Scd2* Th1 cells. (**Figure 3B**). We also found that the positioning of IRF9 binding patterns in sg*Scd2* Th1 cells was close to that of IFNβ-treated Th1 cells (**Figure 3C**). To further confirm the motif sequences of IRF9-bound genes, HOMER tools was used to calculate the enrichment of known motifs. A known motif enrichment analysis clearly showed the high enrichment of the interferon-sensitive response element (ISRE) in both of IFNβ-treated and sg*Scd2* Th1 cells (**Figure 3D**). We also performed a genomic regions enrichment of annotations tool (GREAT) analysis to determine the potential biological significance of IRF9-bound peaks. As expected, the GREAT analysis revealed that in IFNβ-treated Th1 cells, the targets of IRF9 contained significant enrichment of several functional categories, including the term response to IFNβ (**Figure 3E**). We also found that comparable results were obtained from IRF9-bound genes in sg*Scd2* Th1 cells. Importantly, the GREAT analysis revealed that the significant accumulation of genes related to the abnormal interferon level in sg*Scd2* Th1 cells, which are also known to be hallmarks of autoimmune diseases, including systemic lupus erythematosus (SLE) and Sjogren’s syndrome (**Figure 3F**). Altogether, the spontaneous production of type I-IFN induced by the genetic deletion of *Scd2* in Th1 cells augmented the tonic type I-IFN stimulation and subsequent induction of IRF9 binding to genes that contained hallmarks of autoimmune disorders.

### ChIP-seq profiles revealed a unique feature of IRF9 binding in sgScd2 Th1 cells

**Figure 4:**
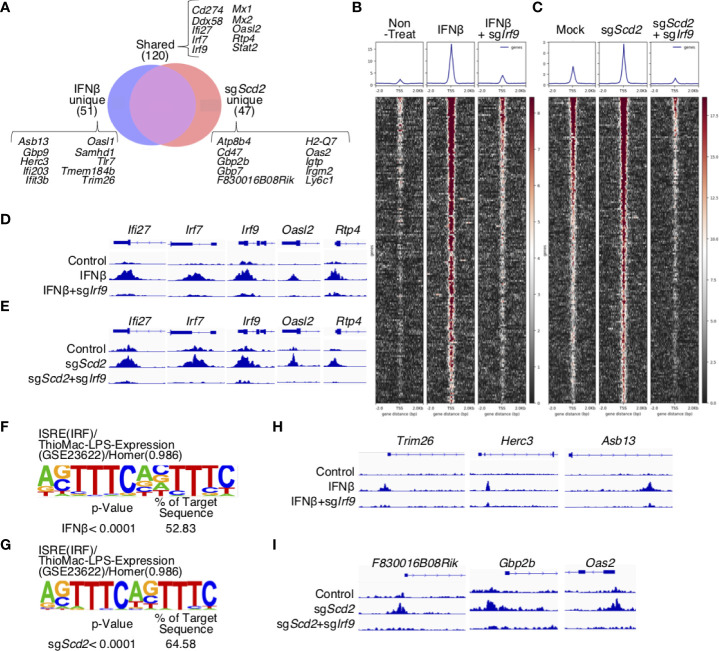
IRF9 in sgScd2 Th1 cells bound to a region that was not bound by IFNβ-treated Th1 cells. **(A)** Venn diagrams showing the peak count for IRF9 ChIP-seq. Peaks were divided into IFNβ-unique, shared and sgScd2-unique sections. **(B, C)** Heatmaps and average aggregate plots were generated using deeptools plotHeatmap and plotProfile in non-treat, IFNβ and IFNβ-treated sgIrf9 **(B)**, or mock, sgScd2 and sgScd2/sgIrf9 Th1 cells **(C)**. TSS from 2kb upstream of the transcriptional start site to -2 kb downstream was analyzed. **(D, E)** Example of shared IRF9 binding sites at the genomic region for Ifi27, Irf7, Irf9, Oasl2 and Rtp4 in non-treat, IFNβ and IFNβ-treated sgIrf9 **(D)** or mock, sgScd2 and sgScd2/sgIrf9 Th1 cells **(E)**. The arrow indicates the direction of transcription. **(F, G)** Results of the HOMER de novo motif analysis from IFNβ-unique peaks **(F)** or sgScd2-unique peaks **(G)** relevant to **(A)**. **(H, I)** Example of IRF9 binding sites at the genomic region for Trim26, Herc3 and Asb13 in IFNβ-unique peaks **(H)** and F830016B08Rik, Gbp2b and Oas2 in sgScd2-unique peaks **(I)**.

We next analyzed ChIP-seq data in greater detail to evaluate the similarities and differences between the IRF9 binding profiles in sg*Scd2* Th1 cells and IFNβ-treated Th1 cells. A considerable percentage of the IRF9 target genes, including *Cd274, Ddx58, Ifi27, Irf7, Irf9, Mx1, Mx2, Oasl2, Rtp4*, and *Stat2*, were shared by *Scd2*-deleted and IFNβ-treated Th1 cells (71.8% and 70.1%, respectively) (**Figure 4A**). We also observed sg*Scd2*-unique IRF9-bound genes, including *Atp8b4, Cd47, Gbp2b, Gbp7, F830016B08Rik* and *Oas2.* Similarly, *Asb13, Gbp9, Herc3, Ifi203, Ifit3b* and *Samhd1*, were observed as IFNβ-unique genes. The deletion of *Irf9* was associated with a very weak enrichment of ChIP-seq peaks in both IFNβ-treated and sg*Scd2* Th1 cells, suggesting that the peaks obtained from our experiments showed IRF9-specific DNA binding regions. According to the profiles, we found that the deletion of *Scd2* efficiently induced IRF9 to bind the transcription start site (TSS) to the same extent as IFNβ-treated Th1 cells (**Figures 4B, C**). We also confirmed that IRF9 binding was observed in the majority of ISGs, including *Ifi27*, *Irf7*, *Irf9*, *Oasl2*, *Rtp4*, in both IFNβ-treated and sg*Scd2* Th1 cells (**Figures 4D, E**). To further investigate IRF9-specific DNA binding regions, we next focused on IFNβ- and sg*Scd2*-unique peaks. A *de novo* motif analysis of IFNβ- and sg*Scd2*-unique peaks revealed significant enrichment of the ISRE consensus binding site in both groups (**Figures 4F, G**). We also found that IFNβ-unique peaks contained *Trim26*, *Herc3*, and *Asb13*, while sg*Scd2*-unique peaks contained *F830016B08Rik*, *Gbp2b*, *Oas2*. In particular, *Trim26*, *Herc3*, *Gbp2b* and *Oas2* are known to be related to the progression of autoimmune disease (**Figures 4H, I** and **Supplementary Figures 4A–D**). Thus, detailed ChIP-seq profiles revealed a unique feature of IRF9 binding in sg*Scd2* Th1 cells that were not observed in IFNβ-treated Th1 cells.

### Integrated analysis of RNA-seq and IRF9 ChIP-seq in sg*Scd2* Th1 cells

**Figure 5:**
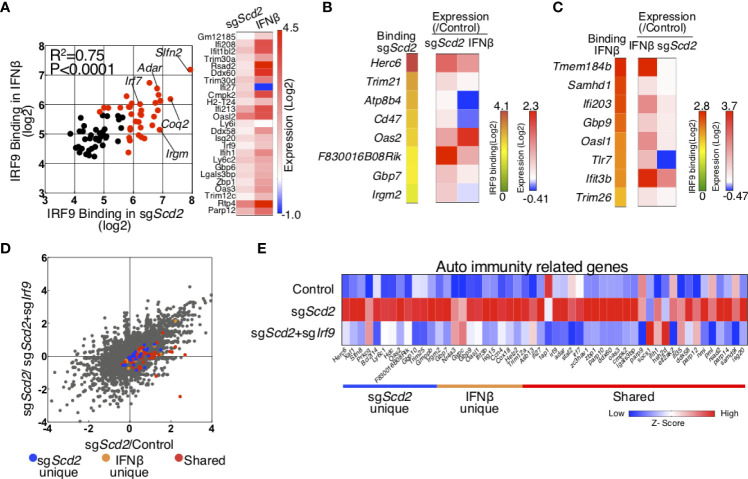
Integrated analysis of RNA-seq and IRF9 ChIP-seq was performed to investigate ISG transcriptome regulated by IRF9. **(A)** Scatter plots depicting IRF9 binding in sgScd2 Th1 cells (x axes) and IFNβ-treated Th1 cells (y axes) for all shared IRF9-bound genes. Genes with signal intensities higher than 5.64 (Log2) are marked with red dots. A heat map shows 1.5-fold upregulated genes in sgScd2 Th1 cells. **(B, C)** A heat map shows normalized IRF9 ChIP-seq signal intensities relevant to **Figure 4A** and relative gene expression using qRT-PCR in sgScd2-unique **(B)** or IFNβ-unique peaks **(C)**. **(D)** A dot plot of gene expression by RNA-sequencing relevant to **Figure 1** compares in sgScd2 Th1 cells/control or sgScd2+ sgIrf9 Th1/control Th1 cells. The genes related to sgScd2-unique, IFNβ-unique peaks or shared were marked with blue, orange or red dots, respectively. **(E)** A heat map shows relative expression of auto immunity related genes in control, sgScd2 and sgScd2/sgIrf9 Th1 cells. Three technical replicates were performed with quantitative RT-PCR **(B, C)**. Three experiments were performed and showed similar results.

To globally determine the genes controlled by IRF9 activated *via* the modulation of MUFA metabolism, we combined transcriptional profiling with genome-wide mapping of IRF9 target genes in sg*Scd2* Th1 cells and IFNβ-treated Th1 cells. To this end, we first focused on the shared IRF9-bound genes and found a strong positive correlation between the levels of IRF9 binding in sg*Scd2* Th1 cells and IFNβ-treated Th1 cells (**Figure 5A**). Consistent with this result, the expression levels of shared ISGs (other than *Ifi27)* were increased in both IFNβ-treated and sg*Scd2* Th1 cells (**Figure 5A**). These data indicate the functional role of IRF9 in the regulation of the ISGs expression in sg*Scd2* Th1 cells. Next, we focused on the sg*Scd2*-unique genes and compared the expression levels of the genes between IFNβ-treated and sg*Scd2* Th1 cells. Even though expression of some genes were increased by IFNβ stimulation, the levels tended to be higher in sg*Scd2* Th1 cells (**Figure 5B**). We also found that the higher expression levels of IFNβ-unique genes in IFNβ-treated Th1 cells (**Figure 5C**). Therefore, these data indicate that IRF9 has a selective functional role in the transcription of genes characterized by IRF9 binding in IFNβ-treated or sg*Scd2* Th1 cells, respectively. To examine the relationship between IRF9-bound genes and their expression levels in more detail, we compared the gene expression profiles of sg*Scd2* and sg*Scd2*/sg*Irf9* Th1 cells. The expression levels of shared genes tended to be strongly upregulated in sg*Scd2* Th1 cells in comparison to *Scd2-*unique and IFNβ-unique genes (**Figure 5D**). We also found that the double knockout of *Scd2* and *Irf9* resulted in the downregulation of IRF9-bound genes that were uniquely detected in or IFNβ-treated or sg*Scd2* Th1 cells. These results show the importance of IRF9 to construct ISG transcriptome in sg*Scd2* Th1 cells. Finally, to investigate the association between the IRF9-regulated ISGs expression in sg*Scd2* Th1 cells and autoimmune diseases, we focused on the expression levels of ISGs that are elevated in patients with SLE (31). A transcriptomic analysis revealed that ISGs related to the pathogenesis of SLE was upregulated in sg*Scd2* Th1 cells in an IRF9-dependent manner (**Figure 5E**). Importantly, the expression levels of IRF9-bound genes that are uniquely regulated by the genetic deletion of *Scd*2 or IFNβ treatment were downregulated by genes targeting *Irf9* to almost the same extent as by shared genes (**Figure 5E**). Taken together, sg*Scd2* Th1 cells activate IRF9 nuclear translocation, resulting in the construction of a unique ISG transcriptome, which includes autoimmune-related ISGs.

## Discussion

In the present study, we discovered that IRF3 and IRF9, but not IRF7, are important in the regulation of SCD2-mediated type I-IFN responses in T cells. Using RNA-seq analyses, the increased expression of ISGs in sg*Scd2* Th1 cells was found to be suppressed by the deletion of *Irf3* or *Irf9*. Although IRF7 has been known to be an essential transcriptional factor for the production of type I-IFN in innate immune cells (32), the genetic deletion of *Irf7* only very slightly affected the ISGs expression in sg*Scd2* Th1 cells. Using ChIP-seq analyses, we investigated the genome-wide binding of IRF9 in sg*Scd2* and IFNβ-treated Th1 cells in detail. A genome-wide comparison of IRF9 occupancy identified 120 shared genes in sg*Scd2* and IFNβ-treated Th1 cells. We also found 47, or 51 unique IRF9-bound genes in sg*Scd2* or IFNβ-treated Th1 cells, respectively. In addition, through a combination of RNA-seq and IRF9 ChIP-seq analyses, we defined the novel functional properties regarding IRF9-mediated regulation of sg*Scd2*-unique, IFNβ-unique and shared IRF9-bound genes. Taken together, our findings provide a novel molecular cascade wherein SCD2-mediated type I-IFN responses require IRF3 to induce type I-IFN production, resulting in the activation of IRF9 to drive ISG transcription.

Many studies on the type I-IFN pathways have been conducted in innate immune cells (2, 5, 10). As a key, in type I-IFN signaling during viral infection, pathogen-derived nucleic acid activates STING and MAVS, which recognize intracellular DNA and RNA, respectively (5). In turn, STING and MAVS activate IRF3 and IRF7 to induce the production of type I-IFN, resulting in the stimulation of downstream anti-viral responses (2, 5). Consistently, we have previously reported that higher amounts of cGAMP and cytosolic DNA originated from genomic DNA in sg*Scd2* Th1 cells, leading to the activation of the STING pathway (18). Secreted type I-IFN plays a critical role in inducing the nuclear translocation of IRF9 in an autocrine (and/or paracrine) fashion in macrophages (7, 33). Upon the binding of type I-IFN to IFNAR, the JAK/STAT pathway is activated to induce the construction of ISGF3—which is composed of STAT1, STAT2 and IRF9—for the transactivation of ISGs (7, 33). We now show that MUFA-mediated STING activation is crucial for IRF3 to produce type I-IFN and subsequent IRF9-dependent transactivation of ISGs. Importantly, a GSEA showed significant enrichment of ISGs in sg*Scd2* Th1 cells. These ISGs, including *Rsad2*, *Ifit3*, *Isg15*, *Xaf1*, *Oas3*, *Isg20, Slfn Ifit2*, *Irf9*, *bst2*, *Ifi36*, *Irf7* and *Trim56*, were also upregulated by type I-IFN stimulation in innate immune cells (34, 35). Thus, consistent with the series of molecular pathways observed in innate immune cells, our present study indicated that sg*Scd2* Th1 cells showed the similar type I-IFN signaling cascade to innate immune cells.

An intriguing finding in the present study is that IRF3, but not IRF7 is critical for the production of type I-IFN in sg*Scd2* Th1 cells. The importance of IRF7 for type I-IFN production in innate immune cells has been well studied (32, 36). Honda et al., reported that IRF7 is crucial for the induction of type I-IFN production in pDCs infected with VSV or HSV (32). Furthermore, the capability of MEFs to produce type I-IFN during VSV or HSV infection is heavily dependent on IRF7 rather than on IRF3. Moreover, some reports have shown that IRF7 is required to induce type I-IFN responses in CD4^+^ T cells (8, 37). For example, cGAMP stimulation of activated CD4^+^ T cells induces IRF3 and IFR7 activation for type I-IFN responses (8). In addition, CD4^+^ T cells from HIV-infected subjects display higher IRF7 phosphorylation in comparison to HIV-free subjects, suggesting the importance of IRF7 in type I-IFN responses in this context (37). It has been reported that IRF7 can be fully activated *via* interaction with MAVS and STING (38). The study suggests that activation of IRF7 is more stringent than the activation of IRF3 (38). Mechanistically, IRF3 only requires phosphorylation of the 2S site for activation, whereas IRF7 requires phosphorylation of both the 2S and 4S sites for activation (38). In relation to the report, we previously showed that MAVS was not involved in type I-IFN responses in sg*Scd2* Th1 cells (18). Furthermore, we now show that the phosphorylation level of IRF7 was unchanged between control and sg*Scd2* Th1 cells. Taken together, it is possible that MAVS-dependent full phosphorylation may be required for the activation of IRF7, which is the reason why IRF7 is not involved in the regulation of type I-IFN responses in sg*Scd2* Th1 cells.

Several studies have shown that type I-IFN responses and type I-IFN associated signaling pathways contribute to the pathogenesis of various autoimmune diseases (2, 5, 6). These studies reported that higher amounts of type I-IFN and the upregulation of ISGs are detected in patients with auto-immune disease, including rheumatoid arthritis, SLE and Sjogren’s syndrome (1). It has been documented that ~15% of a total cohort of 41 patients with SLE showed high amounts of cGAMP in their serum, indicating that the activation of the cGAS-STING pathway could be involved in the pathogenesis (2). Furthermore, the *IRF9* expression level is also elevated in SLE monocytes in comparison to healthy controls, and its expression is associated with enhanced levels of ISGs at baseline in SLE patients (39). Additionally, genes related to type I-IFN activity including, *ICSBP1*, *MX1*, *IFITM1*, *IFITM2*, *IRF9*, were found to be overexpressed in patients with Sjogren’s syndrome (40). Our combined analyses of transcriptome and IRF9 ChIP-seq revealed that the autoimmunity related genes, which increase in patients with SLE were selectively increased in sg*Scd2* Th1 cells. In particular, sg*Scd2*-unique IRF9 bound genes, including *Herc6, Oas2, Xaf1*, were deeply related to autoimmunity and suppressed by the genetic deletion of *Irf9*. Since the deletion of *Scd2* activates the cGAS-STIING axis for IRF3-mediated type I-IFN production and consequently stimulates IRF9-mediated ISG transcription, our findings could be useful to interpret the disease progression and treatment of autoimmune disease.

In conclusion, our results provide mechanistic insight into the regulatory relationship between lipid metabolism and the IRF family members in the type I IFN response. The genetic deletion of *Scd2* augmented the activation of IRF3, but not IRF7, for type I-IFN production to induce nuclear translocation of IRF9. Furthermore, using a combination of IRF9 ChIP-seq and transcriptome analyses, we found the deletion of *Scd2* causes IRF9 binding to unique genes that are not detected in type I-IFN-treated Th1 cells. Thus, the findings presented herein provide evidence of the involvement of lipid metabolism in the regulation of a family of IRF to induce type I-IFN responses and increase the expression of ISGs.

## Data availability statement

The data presented in the study are deposited in the GEO repository, accession number GSE200625 (https://www.ncbi.nlm.nih.gov/geo/query/acc.cgi?acc=GSE200625) and GSE200636 (https://www.ncbi.nlm.nih.gov/geo/query/acc.cgi?acc=GSE200636).

## Ethics statement

The animal study was reviewed and approved by Institution Animal Care and Use Committee of KAZUSA DNA research institute (Registration number:30-1-002).

## Author contributions

TK, and YE conceived and directed the project, designed experiments, interpreted the results, and wrote the paper. TK and YE designed the project, analyzed main experiments. TK, KM, TaN, SY, SS, HA, OO, ToN, and YE developed experimental protocols and performed experiments. All authors contributed to the article and approved the submitted version.

## Funding

This work was supported by grants from the Ministry of Education, Culture, Sports, Science and Technology (MEXT Japan) (Grants-in-Aid: Grant-in-Aid for Scientific Research on Innovative Areas #18H04665, Scientific Research [B]#20H03455, Challenging Research (Exploratory) #20K21618, Early-Career Scientists #21K15476 and Young Scientists (Start-up) #21K20766). The Nakajima Foundation, TERUMO Life Science Foundation, The Tokyo Biochemical Research Foundation, Kato Memorial Bioscience Foundation, The Hamaguchi Foundation for the Advancement of Biochemistry, Suzuken Memorial Foundation, Kanae Foundation for the Promotion of Medical Science, Takeda Science Foundation, Mochida memorial foundation for medical and pharmaceutical research, GSK Japan Research Grant 2019, SENSHIN medical research foundation, Sumitomo foundation, Koyanagi foundation, Kishimoto foundation 2019, Uehara memorial foundation, Nakatomi Foundation, Research foundation for pharmaceutical sciences group A, Cell science research foundation, The Astellas Foundation for Research on Metabolic Disorders, MSD Life Science Foundation, Public Interest Incorporated Foundation, NAGASE Science Technology Foundation, The Canon Foundation, ONO Medical Research Foundation, the Research Grant of the Princess Takamatsu Cancer Research Fund, The Yasuda Medical Foundation and Toray Science Foundation. The funder was not involved in the study design, collection, analysis, interpretation of data, the writing of this article or the decision to submit it for publication.

## Conflict of interest

The authors declare that the research was conducted in the absence of any commercial or financial relationships that could be construed as a potential conflict of interest.

## Publisher’s note

All claims expressed in this article are solely those of the authors and do not necessarily represent those of their affiliated organizations, or those of the publisher, the editors and the reviewers. Any product that may be evaluated in this article, or claim that may be made by its manufacturer, is not guaranteed or endorsed by the publisher.

## Abbreviations

ACC1, Acetyl CoA Carboxylase 1; cGAMP, cyclic GMO-AMP; ChIP-seq, chromatin immunoprecipitation coupled with high-throughput DNA sequencing; CoA, coenzyme A; GREAT, genomic regions enrichment of annotations tool; GSEA, gene set enrichment analysis; HIV, Human immunodeficiency virus; HOMER, Hypergeometric optimization of motif enrichment; HSV, Herpes simplex virus; IFNAR, type I-IFN receptor; IRF, Interferon regulatory family; ISG, interferon stiulated gene; ISGF3, Interferon Stimulated Gene Factor 3; ISRE, interferon-sensitive response element; MUFA, monounsaturated fatty acid; OXPHOS, oxidative phosphorylation; PCA, principal component analysis; pDCs, Plasmacytoid dendritic cells; SCD, Stearyl CoA desaturase; SLE, Systemic lupus erythematosus; TSS, transcription start site; ype I-IFN, Type I interferon; VSV, vesicular stomatitis virus.
