# Supplementary figures and images for: SCD2-mediated cooperative activation of IRF3-IRF9 regulatory circuit controls type I interferon transcriptome in CD4+ T cells
Source: Front Immunol. 2022 Aug 18;13:904875. doi: 10.3389/fimmu.2022.904875 (PMC9436477; doi:10.3389/fimmu.2022.904875)

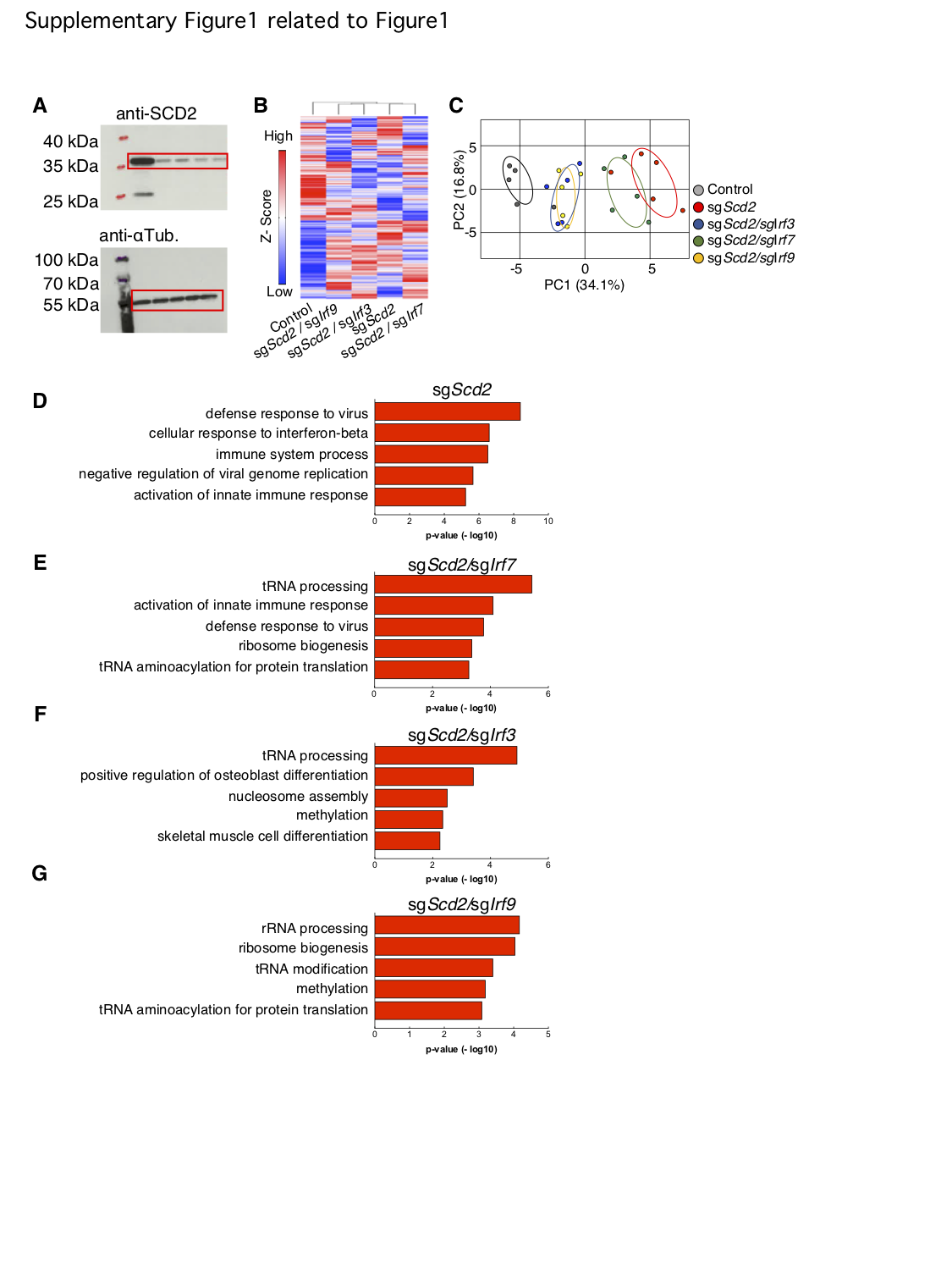

Supplement: Supplementary file 1 [file Image_1.tiff]

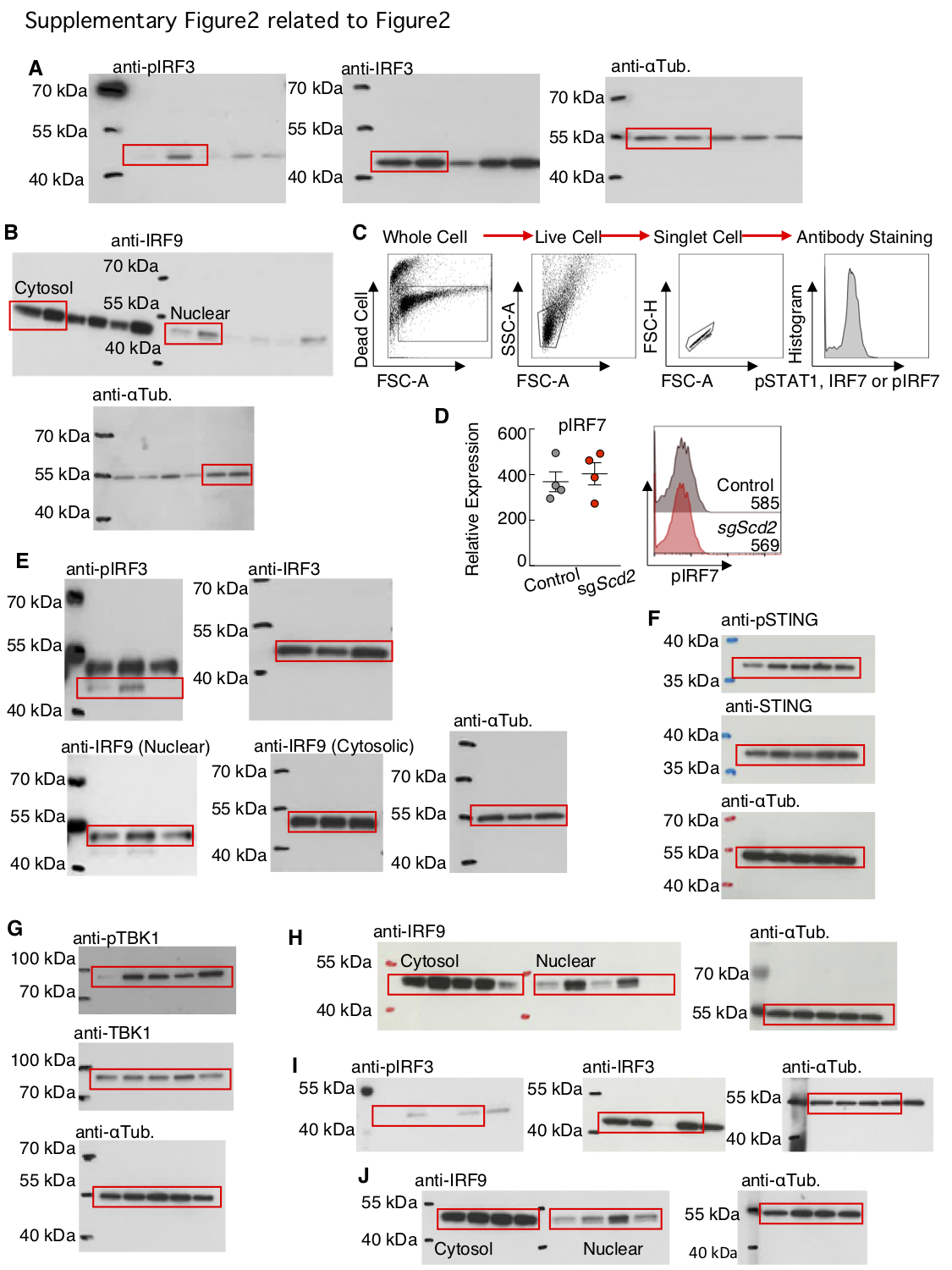

Supplement: Supplementary file 2 [file Image_2.tiff]

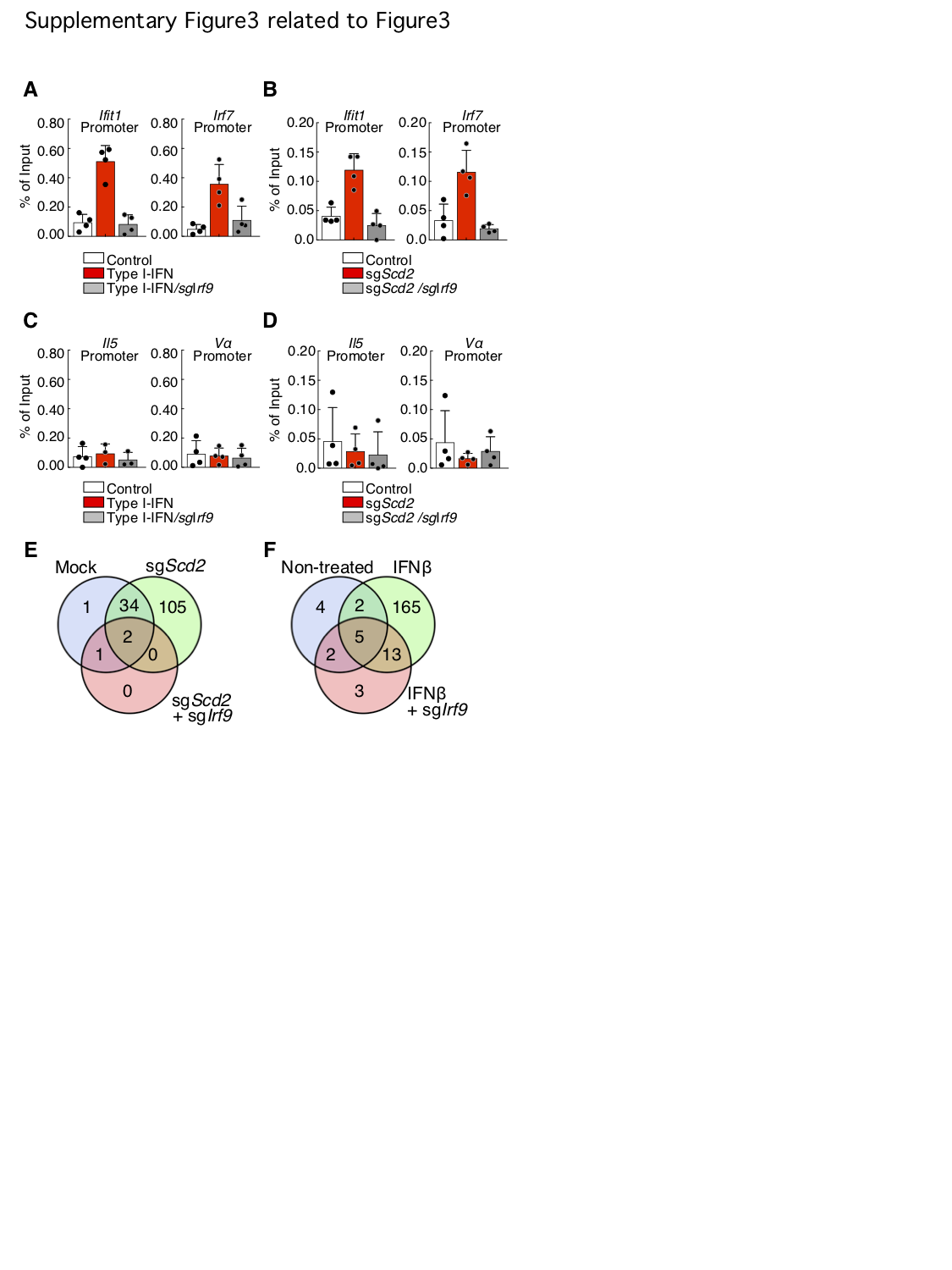

Supplement: Supplementary file 3 [file Image_3.tiff]

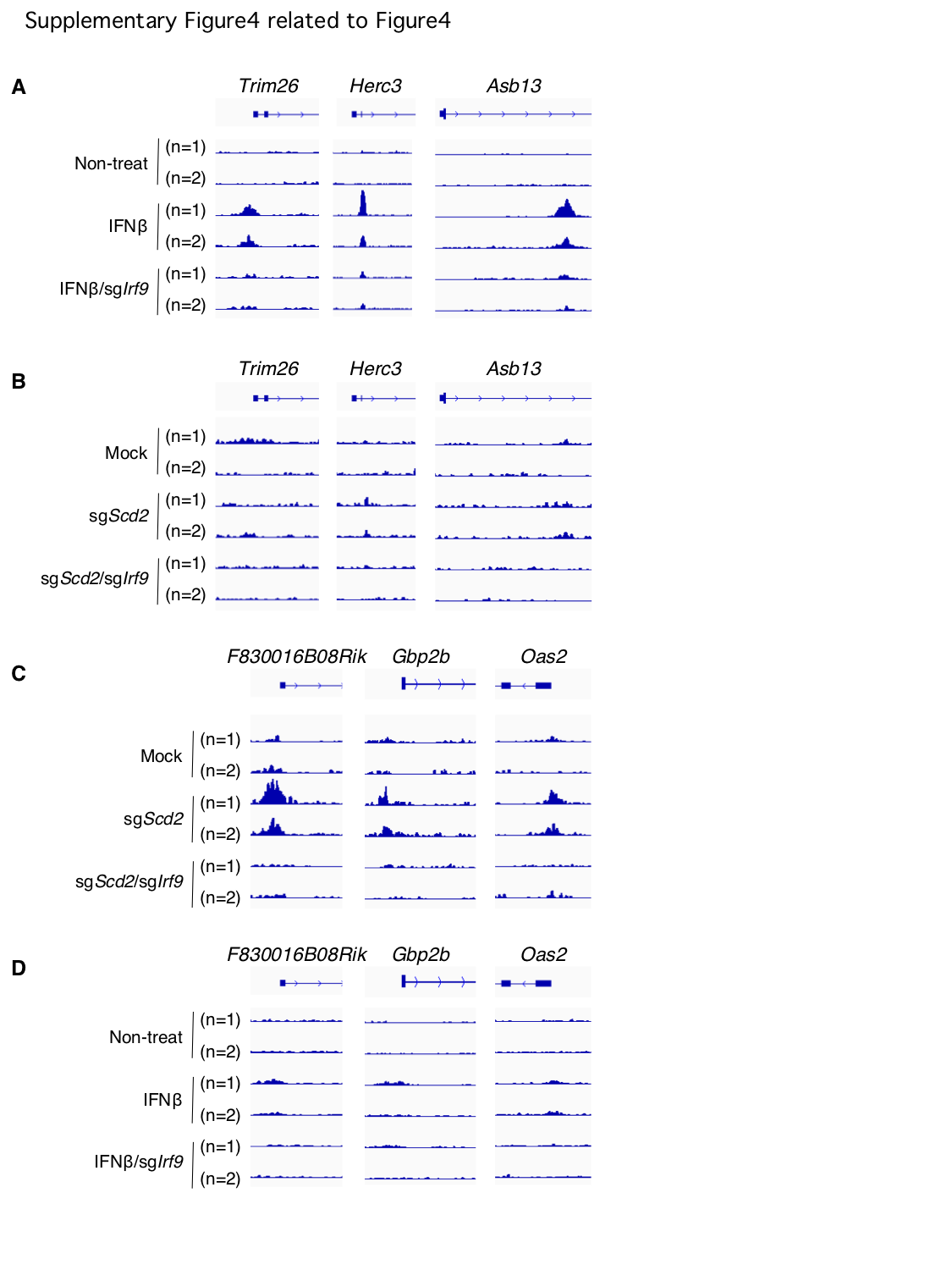

Supplement: Supplementary file 4 [file Image_4.tiff]
